# Supplementary material for: Dependency-aware action planning for smart home
Source: PLoS One. 2024 Jun 18;19(6):e0305415. doi: 10.1371/journal.pone.0305415 (PMC11185447; doi:10.1371/journal.pone.0305415)
Supplement: Attachment — Submitted filename: rebuttal_letter.pdf [file pone.0305415.s002.pdf]

Dependency-Aware Action Planning for Smart Home

Jongjin Kim, Jaeri Lee, Jeongin Yun, and U Kang

We would like to thank the editor for your high quality reviews and constructive comments. Below, we summarize how we addressed the your requirements.

- **(R1)** There are some language corrections in the paper, please proofread carefully.
  - **(A1)** We double-checked the entire manuscript for language errors and made the necessary corrections.
- **(R2)** Provide the source codes related to the papers through GITHUB links.
  - **(A2)** We denote the link of our codes and datasets at the end of introduction section in lines 70 and 81.
- **(R3)** There are several path planning algorithms which are published recently for IoT/WSNs while considering obstacles. The authors must provide them more carefully. For example, Bug2 algorithm-based data fusion using mobile element for IoT-enabled wireless sensor networks, and smart environments related such as Follow-Me AI: Energy-Efficient User Interaction with Smart Environments
  - **(A3)** We add mentioned papers and summarize difference of our work compared to previous works of path planning from lines 580 to 583. Our paper focuses on learning and training an unknown device with its state transition log data, while previous works of path planning aim to effectively control already given smart environments.
- **(R4)** Provide computational complexity for the algorithms 1&2 must be evaluated.
  - **(A4)** We analyze the complexity of algorithms in Complexity Analysis section (line 374). Theorems 1 and 2 analyze the time complexity and the space complexity of Algorithm 1, respectively. Note that we did not separately analyze the complexity of Algorithm 2 which is a subroutine of Algorithm 1.
